# Implicit Racial Attitudes and Associations Among Obstetricians in Hawaiʻi: a Pilot Study

**DOI:** 10.1007/s40615-021-01176-4

**Published:** 2022-01-01

**Authors:** Rebecca Delafield, Andrea Hermosura, Hyeong Jun Ahn, Joseph Keaweʻaimoku Kaholokula

**Affiliations:** 1 Department of Native Hawaiian Health, University of Hawaiʻi, John A. Burns School of Medicine, 677 Ala Moana Blvd., Ste. 1016B, Honolulu, HI 96813 USA; 2 Department of Quantitative Health Sciences, University of Hawaiʻi, John A. Burns School of Medicine, 651 Ilalo St. Medical Education Bldg., Ste 411, Honolulu, HI 96813 USA

**Keywords:** Implicit bias, Obstetricians, Pacific Islander, Hawai‘i, Micronesian

## Abstract

**Introduction:**

Pacific Islanders living in Hawai‘i with ancestral ties to islands in the western Pacific region of Micronesia are common targets of uninhibited forms of prejudice in multiple sectors, including healthcare. Whether the explicit societal-level attitudes toward this group are reflected in implicit attitudes among healthcare providers is unknown; therefore, we designed a pilot study to investigate this question. Our study measures implicit racial bias toward Pacific Islanders from Micronesia among Obstetrician-Gynecologists (OB-GYNs) in Hawai‘i.

**Methods:**

We developed 4 new implicit association tests (IATs) to measure implicit attitudes and associations (i.e., stereotypes) toward Pacific Islanders from Micronesia in 2 conditions: (1) Micronesians vs. Whites and (2) Micronesians vs. Japanese Americans. Participants were practicing OB-GYNs in Hawai‘i. The study was conducted online and included survey questions on demographic and physician practice characteristics in addition to IATs. The primary outcome was the mean IAT *D* score. Associations between IAT *D* scores and demographic and practice characteristics were also analyzed.

**Results:**

Of the 49 OB-GYNs, 38 (77.6%) were female, mean age was 40 years, 29.5% were Japanese, 22.7% were White, and none were from a Micronesian ethnic group. The mean IAT *D* score in the Micronesian vs. White condition (*N* = 29) was 0.181, (SD: 0.465, *p* < 0.05) for the Attitude IAT and 0.197 (SD: 0.427; *p* < 0.05) for the Stereotype IAT.

**Conclusion:**

The findings from this pilot suggest a slight degree of implicit bias favoring Whites over Micronesians within this sample of OB-GYNs and warrant a larger investigation into implicit biases toward this unique and understudied Pacific Islander population.

## Introduction

Recent national events have highlighted the problem of racism as a serious threat to public health in the United States (US) [1–3]. Hawai‘i is often recognized for its racial/ethnic diversity and large number of residents who identify as multiracial, leading many to characterize Hawai‘i as a place of racial harmony. Yet, the state’s racial/ethnic diversity does not negate problems of racism and social hierarchies [4–6]. While there is no clear majority in Hawai‘i, “minority status” is reflected in the socio-economic disadvantage and discrimination experienced by Native Hawaiian and Pacific Islander (NHPI) communities and other marginalized groups [7–9]. Racism, discrimination, and negative stereotypes toward Pacific Islanders, and specifically those with ancestral ties to island nations in Micronesia, are well documented [8, 10–12].

The term “Micronesian” can refer to people from any island within Micronesia, a region in the western Pacific that includes highly diverse islander communities with distinct histories, languages, and customs. The term encompasses individuals who ethnically identify with numerous indigenous Pacific Islander groups, such as Chuukese, Chamorro, Palauan, or Kosraean. In Hawaiʻi, it is commonly used as a racialized identifier to reference people with ancestral ties to three independent island nations, the Federated States of Micronesia, the Republic of the Marshall Islands, and the Republic of Palau, which have formed Compact of Free Association agreements with the US. These agreements allow the US exclusive military rights to these nations, and in exchange allow the citizens of those nations the ability to live, study, and work in the US without a visa. As a result of the agreements, migration from these nations to the US has increased dramatically over the past two decades [13]. In this context, the term “Micronesian” intersects with migrant, indigenous, and racialized identities and experiences, which include being targets of discrimination and prejudice in multiple sectors of society [8].

Similar to other racial minority groups, NHPIs and Micronesian communities specifically experience substantial disparities in health and healthcare outcomes [14–17]. Maternal and perinatal outcomes are no exception [18–22]. For example, rates of cesarean delivery among Micronesian women are significantly higher compared to White women, even after adjusting for medical and sociodemographic confounders [19, 20]. In addition, a study that compared indications for cesarean between Micronesian and White patients found that Micronesian women were over three times more likely to experience a cesarean delivery for more subjective medical indications (e.g., non-reassuring fetal heart tracing and arrest of labor) compared to more objective indications, a finding consistent with similar work examining cesarean delivery among Black women in the US [22, 23]. These studies suggest that research into provider bias may help to clarify variables influencing clinical decision-making in the context of cesarean delivery and help to better understand the racial disparities observed [22, 23].

Scholars and leading health agencies are increasingly recognizing the impact of systemic racism and provider bias on health and health care outcomes [2, 24–28]. However, there is a dearth of evidence on bias and racism toward NHPI, despite their growing population in the US [29]. While the striking and persistent health disparities experienced by racial minority groups, including Pacific Islanders, in the US are likely caused by multiple factors, it is important to consider the role of provider bias on these outcomes. Yet, the ability to understand the role of explicit or implicit racial bias and discrimination in observed health disparities is hindered by the lack of assessment instruments of bias toward Pacific Islander communities. Our pilot study aim was to measure implicit racial bias toward Micronesians among OB-GYNs in Hawai‘i. We expected to find that despite the relatively recent establishment of Micronesian as a racialized identity in Hawaiʻi, the test would demonstrate implicit bias that favored non-Micronesian groups.

## Methods

We conducted an online pilot study that measures the implicit attitudes and associations of OB-GYNs in Hawai‘i with salient racial/ethnic groups: Micronesians compared to Whites and Japanese Americans. This pilot was designed to inform the development and implementation of a full-scale study by providing data on the concept (i.e., implicit bias toward Micronesians) and feasibility (i.e., recruitment of physicians for this type of online research). Ethical approval for this study was granted by the Institutional Review Board of the University of Hawai‘i (protocol ID no.: 2017–00,139).

OB-GYN and OB-GYN residents in Hawai‘i were recruited via e-mail between October and December 2018. Approximately 200 OB-GYNs were contacted about the study through professional networks and OB-GYN faculty with the University of Hawai‘i who assisted with recruitment efforts. The availability of demographic data on the OB-GYN workforce was limited, but a 2018 report noted that 61% of actively practicing OB-GYNs in the state were female and 30% were 60 years old or older [30]. The recruitment e-mails contained a secure link that took participants directly to the study website that hosted the survey and IATs. To be eligible for the study, OB-GYNs had to be actively practicing in Hawai‘i, 18 years old or older, and physically able to sit for 40 min to take an online survey. Project Implicit ®, a non-profit organization, research collaboration, was contracted to host and manage the website through secure servers at Harvard University [31]. Participants were given the option to provide an e-mail address (on a separate website so that results were not linked to contact information) to receive a $10 e-gift card for their time.

### Demographic and Practice Measures

We collected information on age, gender, racial/ethnic identity, place and length of residence in Hawai‘i, and whether respondents were born in Hawai‘i. The questions on place of birth (“Were you born in Hawai‘i?”) and length of time in Hawai‘i were collected to understand the length of exposure to the socio-cultural context within the state. The survey also included questions about the respondent’s peer group and practice setting context. Information on estimated (by percentage) racial/ethnic composition of peer groups and the patient panel was collected for the OB-GYN respondents in regard to the three racial/ethnic groups of interest in the study. Participants were asked to select a primary practice setting to indicate where they engaged most with their patients, if they accept Medicaid/Med-QUEST insurance (Med-QUEST is Hawai‘i’s managed care program for Medicaid eligible patients), and if they were currently in a training program (i.e., residency or fellowship). Participants were also asked if they had attended implicit bias training(s) in the past and the number of implicit association tests they had completed (excluding the two from the current study).

### Implicit Bias Measures

The implicit association test (IAT) is a widely utilized and validated measure of implicit bias [31–35]. The IAT is a computer-administrated sorting task that is designed to measure how quickly the participant sorts with two opposing values (e.g., good vs bad terms) to two distinct target concepts (e.g., race A or race B) under different conditions. The IAT is frequently used in psychology and social science research and has been found to have good reliability compared to other implicit bias measures; has convergent validity with other measures; and has moderate predictive validity among adult participants across various target concepts (e.g., race, political preference) [34, 36, 37].

We adapted four IATs to measure implicit bias toward Micronesians compared to Whites and Japanese Americans. The tests for this study measure differences in “attitudes” or attitudinal (“good” vs “bad”) associations and differences in the level of association with the stereotype “dependent” as compared to “independent.” The tests tested differences in associations between Micronesians and Whites (Comparison 1) and Micronesians and Japanese Americans (Comparison 2).

The stimuli for the target categories were carefully selected for each IAT. For the attitude IATs, we utilized stimuli used by Project Implicit for their publicly available IATs [31]. The exemplars for the racial/ethnic target categories were developed by the study team and tested through unstructured and structured sorting tasks. The terms used for all the tests can be found in Table 1. Study team members pre-tested the IATs before the final version was released.

**Table 1:** Targets and stimuli used in IATs

Concepts/targets	Exemplars
Race^a^	
Micronesian	Oceania, Islander, Micronesia, Micronesian
Caucasian	White, Caucasian, European, Blonde
Japanese American	Japanese, Karate, Sushi, Origami
Attitudes	
Good	Joy, Love, Peace, Wonderful, Pleasure, Glorious, Laughter, Happy
Bad	Horrible, Terrible, Agony, Nasty, Evil, Awful, Failure, Hurt
Stereotypes	
Independent	Independent, Productive, Self-sufficient, Contributor, Employed
Dependent	Dependent, Freeloader, Reliant, Leech, Unemployed

^a^The term “Caucasian” rather than “White” was selected for the purposes of the test because of the length of the term in relation to other categories. The authors use White throughout the rest of the paper

The target concepts and terms for the stereotype IATs were identified through a formative research study. We used a two-step qualitative research approach to identify primary stereotypes for Micronesians, Japanese Americans, and Whites. The “primary” stereotype was defined as the stereotype identified as the most commonly associated with each group based on the responses from respondents. Step 1 involved an inductive approach to generate information on negative stereotypes using a survey with open-ended questions to solicit a set of presumed negative stereotypes for each group from identified “Experts,” defined as people with expertise in racial/ethnic studies or in healthcare in Hawai‘i. For Step 2, the categories yielded from Step 1 were presented to a small group of graduate-level university students, faculty, or staff in health-related fields using an approach that included facilitated group discussion to review, revise, and eventually, rank the concepts based on a modified nominal group technique [38]. The process identified the primary negative stereotype of Micronesians as “dependent or burden”; therefore, this concept was included in the new IAT. This concept did not overlap with negative stereotypes identified for the comparison groups.

### Explicit Bias Measures

Explicit bias measures were used to evaluate conscious attitudes associated with the target concepts. The measures included two items that require participants to select a statement indicating their comparative degree of preference (that included neutral) between (1) Whites and Micronesians and (2) Japanese Americans and Micronesians. Participants were also asked to indicate their feelings of “warmth” (on a 7-point scale from least warm to most warm) toward the three racial/ethnic groups using a feeling thermometer scale, a measure commonly used to measure the direction and magnitude of an attitude.

The final 11 questions of the survey addressed provider beliefs relating to cesarean delivery, which is not the focus of this analysis and therefore will be reported on elsewhere. The full survey can be found in Online Resource 1.

### Data Collection

Participants accessed the study via a link to a secure website contained in their e-mail. The landing page for the site showed the study informed consent details. Participants indicated their consent by advancing to the next page to initiate the study. Participants were automatically randomized to receive either comparison 1 or comparison 2 and asked to complete two IATs (an attitudinal and stereotype IAT) for that comparison group. The survey items were divided so that the questions about a peer group and implicit bias training and the explicit bias measures were asked after the IATs were completed.

### Analysis

Descriptive statistics of demographic and practice characteristics were analyzed using means for continuous variables and frequencies for categorical variables. The IAT *D* score was calculated using the standard procedures outlined by previous researchers [33]. To determine whether the mean IAT *D* scores for each test were significantly different from zero, we used a one-sample *t*-test and also calculated the effect size (Cohen’s *d*). Pearson’s correlation coefficient (*r*) was used to assess the association between implicit bias scores, explicit bias scores, and demographic and practice measures for continuous variables. To characterize the relationship between categorical variables and IAT *D* scores, we ran a one-way ANOVA. Analyses were conducted using SAS software (version 9.4).

## Results

### Characteristics

Sixty-four participants enrolled in the study, roughly a response rate of 32%. Of the 64 enrolled, 15 (23.4%) failed to complete the survey at various points after advancing past the screening questions. Our final sample size was 49 participants, approximately 25% of those contacted via e-mail. Table 2 provides details on the participant and practice characteristics.

**Table 2:** Participant and practice characteristics (*N* = 49)

Characteristic	*N* (%)	Mean *(range)*
Age		39.9 *(27–83)*
Gender Female	38 (77.6%)	
Race (primary)*		
White	10 (22.7%)	
Japanese	13 (29.5%)	
Chinese	11 (25.0%)	
Native Hawaiian or Black	4 (9.1%)	
Other	11 (25.0%)	
Born in Hawai ‘i Yes	19 (38.8%)	
Years of residence in Hawai‘i		21.4 *(*< *1–75)*
Island of residence Oʻahu	47 (95.9%)	
Estimate of peer population		
White		32.2% (2*–*95%)
Japanese American		38.3% (1*–*95%)
Micronesian		1.1% (0*–*10%)
Currently in training Yes	23 (46.9%)	
Years practiced in Hawai‘i		10.3 *(*< *1, 49)*
Primary practice setting		
Hospital	29 (59.2%)	
Private practice (individual)	10 (20.4%)	
Private practice (group)	6 (12.2%)	
FQHC	4 (8.2%)	
Other	1 (2.0%)	
Accepts Medicaid Yes	44 (89.8%)	
Estimate of patient population		
White		20.1% (1*–*50%)
Japanese American		24.4% (0*–*90%)
Micronesian		28.6% (0*–*95%)
Previous training on implicit bias		
Yes	31 (63.3%)	
No	13 (26.5%)	
I do not know	5 (10.2%)	
Number of IATs taken previously		
0	16 (32.7%)	
1–2	15 (30.6%)	
≥ 3	18 (36.7%)	

*Primary race was established by asking participants what race/ethnicity they “most identified with.” The “Other” category includes any single race category not listed or any multiracial participant that declined to select a “primary race” category

### Implicit and Explicit Measures

Mean IAT *D* scores and effect size for each test are found in Table 3. Results for Micronesian/White comparisons for both the Attitude and the Stereotype IATs indicated a slight pro-White/anti-Micronesian bias based on commonly used cut-off criteria and were statically significant (*p* < 0.05). The results of the Micronesian/Japanese American IATs did not reach significance, possibly due to the slightly smaller sample size (*N* = 21). The implicit bias scores for the Micronesian/White Attitude and Stereotype tests were significantly correlated (*r* = 0.50; *p* = 0.006).

**Table 3:** Mean IAT *D* score and effect size of each implicit association test (IAT) for each comparison condition^a^

IATs	Number	Mean IAT *D*^b^	SD	Min, Max IAT D	Effect size^c^	*p*-value
Micronesian/White attitude	29	0.181	0.465	(− 0.795, 1.376)	0.389	0.045*
Micronesian/White stereotype	29	0.197	0.427	(− 0.606, 1.362)	0.465	0.018*
Micronesian/Japanese attitude	21	0.190	0.448	(− 0.626, 1.171)	0.424	0.066
Micronesian/Japanese stereotype	21	− 0.034	0.362	(− 0.503, 0.874)	0.094	0.670

*p < 0.05

^a^Participants were randomized to one of two conditions: (A) Micronesian/White comparisons (*N* = 29) or (B) Micronesian/Japanese American (*N* = 21). In each condition, two IATs were administered one measuring attitudes and the other measuring association with the stereotype independent/dependent

^b^IAT *D* scores range from − 2 to + 2. A positive mean indicates some preference for Whites or Japanese Americans (depending on the test condition and breakpoints for IAT *D* scores are 0.15–0.34 = slight, 0.35–0.64 = moderate, 0.65 or above = strong preference for Whites or Japanese Americans. A negative mean indicates some preference for Micronesians

^c^Effect size is measured using Cohen’s *d* score ranging from − 1 to + 1

Explicit measures did not suggest bias among the sample, with scores being close to neutral (neutral score is 4) for comparisons between White and Micronesians (mean = 4.31; SD = 1.0) and Japanese Americans and Micronesians (mean = 4.61, SD = 1.0). The scores indicating the level of warmth (1–7 with 7 being most warm) for Whites and Micronesians were similar (mean = 4.90, SD = 1.4 and mean = 5.00, SD = 1.4 respectively), with warmth for Japanese Americans reported as slightly higher (mean = 5.40, SD = 1.4). There was no significant association between implicit bias scores and explicit bias scores.

### Associations Between Characteristics and IAT Scores

Significant associations were found between several demographic characteristics and participant’s IAT *D* scores. Older age, male gender, length of residency in Hawai‘i, and the number of years working in Hawai‘i were significantly and positively associated with IAT *D* scores of both the Attitude IAT and Stereotype IAT for the Micronesian/White comparison (see Table 4).

**Table 4:** Pearson’s correlation coefficient (*r*) between participant and practice characteristics and IAT scores

Characteristics (*N* = 29)	Micronesian/White Attitude IAT	Micronesian/White Stereotype IAT
Age	0.64**	0.55**
Length of residency in Hawai‘i	0.55**	0.47*
Length of time working in Hawai‘i	0.62**	0.54**
Percent of peer group^a^ that is Japanese American	0.26	0.33
Percent of peer group^a^ that is Micronesian	0.11	− 0.19
Percent of peer group^a^ that is White	− 0.06	− 0.15
Percent of patient population that is Japanese American	0.40*	0.36
Percent of patient population that is Micronesian	− 0.36	− 0.23
Percent of patient population that is White	0.04	− 0.26

*p < 0.05; **p < 0.001

^a^1 missing value, *N* = 28

There were also significant associations between IAT *D* scores and practice characteristics. We found that participants who reported they were in training (e.g., residents or fellows) had significantly lower IAT *D* scores on average compared to those who were not in a training program. There was a significant difference in IAT *D* scores for the Micronesian/White Attitude IAT by the provider type (Table 5), with lower pro-White bias among providers who reported the majority of their interaction with patients at the hospital.

**Table 5:** Association between IAT score and gender, training, and provider settings

IATs	Groups	Number	Mean IAT *D*^*a*^	Std Dev	Effect size^b^	*p*-value
Association between **Gender** and IAT scores						
Micronesian/White attitude	Female	21	0.048	0.415	0.116	0.026*
	Male	8	0.529	0.426	1.243	
Micronesian/White stereotype	Female	21	0.182	0.356	0.510	0.900
	Male	8	0.243	0.603	0.403	
*p < 0.05						
Association between **Training** and IAT scores						
Micronesian/White attitude	In training	11	− 0.132	0.376	0.350	0.005*
	Not in training	18	0.372	0.414	0.898	
Micronesian/White stereotype	In training	11	0.054	0.378	0.144	0.280
	Not in training	18	0.287	0.441	0.650	
*p < 0.05						
Association between **ProviderSetting** and IAT scores						
Micronesian/White attitude	Hospital	14	− 0.10	0.43	0.233	0.011*
	Private Individual	6	0.52	0.46	1.130	
	Private group	5	0.40	0.26	1.538	
	FQHC^c^	3	0.48	0.13		
Micronesian/White stereotype	Hospital	13	0.04	0.36	0.111	0.380
	Private individual	6	0.50	0.53	0.943	
	Private group	6	0.23	0.31	0.742	
	FQHC^c^	3	0.30	0.59		

*p < 0.05 based on Kruskal Willis test to account for sample size differences

^a^A positive IAT *D* score indicates some preference for Whites and breakpoints for IAT *D* scores are 0.15–0.34 = slight, 0.35–0.64 = moderate, 0.65 or above = strong preference. A negative mean indicates some preference for Micronesians

^b^Effect size is measured using Cohen’s *d* score ranging from − 1 to + 1

^c^FQHC stands for Federally Qualified Health Center. Note: Effect size is compared to mean = 0

## Discussion

To our knowledge, this study is the first to measure implicit and explicit racial/ethnic bias among physicians in Hawai‘i. The results of this pilot investigation suggest that among this small sample of practicing OB-GYNs in Hawai‘i, there is a slight pro-White/anti-Micronesian bias when measuring associations with both attitudes and the stereotype dependent (vs independent). Results for the Micronesian/Japanese American condition were not statistically significant.

Higher IAT *D* scores were significantly associated with multiple demographic and practice characteristics, but not correlated with the race/ethnicity of the physician. The associations we found between mean IAT *D* scores and gender and age were also noted in several other studies that examined implicit attitudes about race among physicians [30–32]. Implicit and explicit bias measures were not significantly correlated in this study, which is consistent with previous studies and may be explained in part by the controlled vs automatic nature of explicit compared to implicit biases, especially in situation where self-report options include responses that are generally more socially acceptable than others [34, 39, 40].

We found a pro-White bias among physicians in this study, which is consistent with other research on implicit bias among healthcare providers [39, 41–43]. The overall level of pro-White bias among our sample was lower than reported in other studies that measured implicit bias among medical professionals who took IATs comparing Black faces and White faces [39–41]. In a large sample of test-takers that voluntarily took the Race Attitude test (comparing Black and White faces) IAT on the Project Implicit website, Sabin et al. (2009) found a mean IAT *D* score of 0.39 (SD 0.47; Cohen’s *d* = 0.89) [41]. A smaller study that enrolled pediatric faculty, fellows, and residents found a mean IAT *D* score of 0.18 (*p* ≤ 0.01; SD 0.44; Cohen’s *d* = 0.41) on the Black/White Race Attitude test [39]. A separate study that developed an IAT to compare attitudes toward Native Americans and Whites reported a mean IAT *D* score indicating a slight pro-White preference (mean IAT *D* = 0.19; range − 1.93 to 1.16; Cohen’s *d* = 0.35) [42].

The lower scores observed in our study could be influenced by several factors, but we briefly explore two here: (1) social context and exposure to negative messaging and (2) bias in our convenience sample. A difference observed when comparing study results from a Black/White comparison and a Micronesian/White comparison may be explained by differences in social context. Perhaps most pertinent to the issue of implicit bias is that negative societal messaging and stereotypes directed at Blacks are widespread and persistent in our US society, while negative stereotypes and messages directed at the specific racialized identity of Micronesians is generally less common. The positive correlation between higher IAT *D* scores and longer length of time living in Hawai‘i is congruent with the idea that longer exposure to racialized messages that associate Micronesian identity with specific attitudes/stereotype may increase implicit racial biases.

One limitation of this pilot study was the use of a convenience sample. We speculate the results may be influenced by the overrepresentation of trainees and females within our sample. We found these characteristics were associated with lower IAT *D* scores on average. Previous studies have also found lower IAT *D* scores among females compared to males [41]. Additionally, it is likely that status as a trainee overlaps with younger age, shorter time living, and working in Hawai‘i, all traits that tended to have lower IAT *D* compared to their counterparts. These aspects may have skewed our results to a more conservative estimate of bias in the population than we would have found in a random sample, possibly resulting in an underestimate of pro-White/anti-Micronesian bias.

Another limitation to note is that our small sample size hindered our ability to conduct a multivariate analysis, which may have helped us gain a clearer picture of how implicit bias differs when controlling for covariates (e.g., age or training status). While the sample size was small, many of our results reached significance, suggesting that a larger study could reveal similar patterns with more power to examine the relationships between variables, adjusting for potential confounding factors.

Because this is a pilot for a larger investigation, analyzing the response and retention is helpful for informing the design of the next study. Our estimated initial response rate was approximately 32%, but the number of responses we could include in our analysis was lower because 15 participants failed to complete the survey. A study investigating response rates to an e-mailed survey among physicians in Canada by specialty (obstetrics was not included) found a 35% overall response rate, but a lower rate among surgeons (30%) and pediatricians (29%) [44]. Our lower response rate may have reflected factors identified in other research including the time demands on clinicians, perceived irrelevance of the survey, or some dissatisfaction with/sensitivity to the survey questions or topic [44, 45]. A future study should consider revisions to the recruitment design recommended by other researchers such as increasing the incentive, reaching out through direct peer contact, or timing the survey to coincide with relevant trainings [44, 45].

There are three aspects of these findings that are important contributions to the literature. First, this is the first study to measure implicit bias toward a Pacific Islander group within the US. Second, we identified bias within a sample that was majority non-White suggesting that diversity in and of itself does not expunge problems of racial implicit bias. And lastly, populations not typically identified in standard racial categories, but who have been “racialized,” are subjected to implicit biases. The findings from this pilot study suggest a larger research study could help us more clearly and comprehensively understand the level of implicit bias within OB-GYNs and how provider characteristics might influence bias. Furthermore, future studies could reveal what interventions are effective at reducing bias, and, critically, whether implicit bias has an impact on patient care for Micronesian women in Hawai ‘i.

## Conclusion

In this pilot investigation of a new IAT tailored to Hawai‘i’s social context, we found a slight degree of pro-White compared to Micronesian bias among the physicians in our sample. Our results indicated similar patterns of bias that favor a White versus a racial minority group and similar characteristics associated with bias, such as age and gender seen in other research studies that examine implicit bias as measured by IATs.

## Data Availability

Data may be made available upon reasonable request to the corresponding author.
